# Dexamethasone therapy versus surgery for chronic subdural haematoma (DECSA trial): study protocol for a randomised controlled trial

**DOI:** 10.1186/s13063-018-2945-4

**Published:** 2018-10-20

**Authors:** Ishita P. Miah, Dana C. Holl, Wilco C. Peul, Robert Walchenbach, Nyika Kruyt, Karlijn de Laat, Radboud W. Koot, Victor Volovici, Clemens M. F. Dirven, Fop van Kooten, Kuan H. Kho, Heleen M. den Hertog, Joukje van der Naalt, Bram Jacobs, Rob J. M. Groen, Hester F. Lingsma, Ruben Dammers, Korné Jellema, Niels A. van der Gaag, on behalf of the Dutch Subdural Hematoma Research Group (DSHR)

**Affiliations:** 1 Department of Neurology and Neurosurgery, Haaglanden Medical Centre (HMC), Lijnbaan 32, 2512 VA The Hague, The Netherlands; 2 Department of Neurology and Neurosurgery, Erasmus Medical Centre (EMC), Dr. Molewaterplein 40, 3015 GD Rotterdam, The Netherlands; 3 Department of Public Health, Erasmus Medical Centre (EMC), Dr. Molewaterplein 40, 3015 GD Rotterdam, The Netherlands; 4 Department of Neurology and Neurosurgery, Leiden University Medical Centre (LUMC), Albinusdreef 2, 2333 ZA Leiden, The Netherlands; 5 Department of Neurology and Neurosurgery, Haga Teaching Hospital, Els Borst-Eilersplein 275, 2545 AA The Hague, The Netherlands; 6 Department of Neurosurgery, Medisch Spectrum Twente (MST), Koningsplein 1, 7512 KZ Enschede, The Netherlands; 7 Department of Neurology, Isala Hospital Zwolle, Dokter van Heesweg 2, 8025 AB Zwolle, The Netherlands; 8 Department of Neurology and Neurosurgery, University of Groningen, University Medical Centre Groningen (UMCG), Hanzeplein 1, 9713 GZ Groningen, The Netherlands

**Keywords:** Dexamethasone, DXM, Chronic subdural haematoma, CSDH, Burr-hole craniostomy, BHC

## Abstract

**Background:**

Chronic subdural haematoma (CSDH) is a common neurological disease with a rapidly rising incidence due to increasing age and widespread use of anticoagulants. Surgical intervention by burr-hole craniotomy (BHC) is the current standard practice for symptomatic patients, but associated with complications, a recurrence rate of up to 30% and increased mortality. Dexamethasone (DXM) therapy is, therefore, used as a non-surgical alternative but considered to achieve a lower success rate. Furthermore, the benefit of DXM therapy appears much more deliberate than the immediate relief from BHC. Lack of evidence and clinical equipoise among caregivers prompts the need for a head-to-head randomised controlled trial. The objective of this study is to compare the effect of primary DXM therapy versus primary BHC on functional outcome and cost-effectiveness in symptomatic patients with CSDH.

**Methods/Design:**

This study is a prospective, multicentre, randomised controlled trial (RCT). Consecutive patients with a CSDH with a Markwalder Grading Scale (MGS) grade 1 to 3 will be randomised to treatment with DXM or BHC. The DXM treatment scheme will be 16 mg DXM per day (8 mg twice daily, days 1 to 4) which is then halved every 3 days until a dosage of 0.5 mg a day on day 19 and stopped on day 20. If the treatment response is insufficient (i.e. persistent or progressive symptomatology due to insufficient haematoma resolution), additional surgery can be performed. The primary outcomes are the functional outcome by means of the modified Rankin Scale (mRS) score at 3 months and cost-effectiveness at 12 months. Secondary outcomes are quality of life at 3 and 12 months using the Short Form Health Survey (SF-36) and Quality of Life after Brain Injury Overall Scale (QOLIBRI), haematoma thickness after 2 weeks on follow–up computed tomography (CT), haematoma recurrence during the first 12 months, complications and drug-related adverse events, failure of therapy within 12 months after randomisation and requiring intervention, mortality during the first 3 and 12 months, duration of hospital stay and overall healthcare and productivity costs. To test non-inferiority of DXM therapy compared to BHC, 210 patients in each treatment arm are required (assumed adjusted common odds ratio DXM compared to BHC 1.15, limit for inferiority < 0.9). The aim is to include a total of 420 patients in 3 years with an enrolment rate of 60%.

**Discussion:**

The present study should demonstrate whether treatment with DXM is as effective as BHC on functional outcome, at lower costs.

**Trial registration:**

EUCTR 2015-001563-39. Date of registration: 29 March 2015.

**Electronic supplementary material:**

The online version of this article (10.1186/s13063-018-2945-4) contains supplementary material, which is available to authorized users.

## Background

A chronic subdural haematoma (CSDH) is a common neurological disease with a rapidly rising prevalence due to increasing age and the widespread use of anticoagulants [1–4]. It generally affects the elderly population and patients with coagulopathy, who often have co-existing medical diseases [1, 5]. The estimated incidence in Western countries is 8.1 per 100,000 per year in patients aged 65 years or older [6], but increases to 58/100,000/year for those aged 70 years or older [1, 7].

Surgical intervention by burr-hole craniotomy (BHC) followed by subdural drainage is the mainstay treatment in symptomatic patients with a CSDH [8, 9], which leads to a favourable functional outcome in 84% of patients [10]. However, despite the optimisation of techniques surgery is still associated with relevant complications, recurrence rates up to 30%, and considerable mortality [8–12]. In addition, especially advanced age and the presence of comorbidities could render patients ineligible for BHC.

Dexamethasone (DXM) therapy has been proposed as an alternative, non-operative or adjuvant treatment modality and might have the potential to block the anti-inflammatory changes in the formation of the haematoma and can specifically impede the formation of neo-membranes and neo-capillaries by its powerful inhibition of inflammatory mediators [13, 14]. Therefore, DXM is administered routinely in various institutions.

To date, only three retrospective and one prospective study have compared the clinical effect of DXM to BHC in CSDH patients [15]. To date, no randomised trials have been published that compare both treatments. Therefore, we designed the DECSA trial: a randomised controlled, multicentre trial to evaluate the non-inferiority of primary DXM compared to primary BHC on functional outcome and cost-effectiveness in patients with symptomatic CSDH.

## Methods/Design

### Trial design

This is a prospective, multicentre, open-label, randomised controlled trial (RCT) with a blinded endpoint (PROBE design) assessment [16]. Eligible patients are randomised to DXM therapy (the intervention arm) or BHC (control arm; see Additional file 1 for SPIRIT check-list).

### Primary study objective

The primary objective is to evaluate the non-inferiority of primary DXM therapy versus primary BHC on functional outcome as expressed by modified Rankin Scale (mRS) score (Table 1) at 3 months and cost-effectiveness at 12 months in patients with symptomatic CSDH.

**Table 1:** Modified Rankin Scale (mRS)

Score	Functional status
0	No symptoms
1	No significant disability. Able to carry out all usual activities despite some symptoms
2	Slight disability. Able to look after own affairs without assistance, but unable to carry out all previous activities
3	Moderate disability. Requires some help, but able to walk unassisted
4	Moderately severe disability. Unable to attend to own bodily needs without assistance, and unable to walk unassisted
5	Severe disability. Requires constant nursing care and attention, bedridden, incontinent
6	Dead

### Secondary objectives

The secondary objectives of the study are functional and clinical outcome, expressed by mRS and Markwalder Grading Scale (MGS) scores (Table 2), respectively, at discharge, at 2 weeks, 3, 6 and 12 months and Glasgow Outcome Scale-Extended (GOSE) score (Table 3) at 3 months. Furthermore, assessment of quality of life using the Short Form – 36 Health Survey (SF-36) and Quality of Life after Brain Injury Overall Scale (QOLIBRI) will take place at 3 and 12 months and healthcare and productivity costs at 3 and 12 months. Haematoma thickness will be evaluated after 2 weeks on follow-up computed tomography (CT). Mortality will be evaluated during the first 3 and 12 months. During the total follow-up period of 12 months we will also evaluate haematoma recurrence, complications and drug-related adverse events, failure of therapy after randomisation and requiring intervention, duration of hospital stay and overall healthcare and productivity costs.

**Table 2:** Markwalder Grading Scale

Score	Clinical status
0	Patient neurological normal
1	Patient alert and oriented; mild symptoms such as headache; absent or mild neurological deficit such as reflex asymmetry
2	Patient drowsy (defined as Glasgow Coma Scale (GCS) score: 13–14) or disoriented with variable neurological deficit, such as hemiparesis
3	Patient stuporous (defined as GCS 9–12) but responding appropriately to noxious stimuli; severe focal signs such as hemiplegia
4	Patient comatose (GCS 8 or lower) with absent motor responses to painful stimuli; decerebrate or decorticate posturing

**Table 3:** Glasgow Outcome Scale-Extended

Score	Category
1	Death
2	Vegetative state
3	Severe disability, lower
4	Severe disability, upper
5	Moderate disability, lower
6	Moderate disability, upper
7	Good recovery, lower
8	Good recovery, upper

### Study setting and participants

Patients will be recruited for the study from the emergency department, neurological or neurosurgical outpatient clinic or ward or through referral from general hospitals of the seven participating Dutch neurosurgical hospitals. The seven participating neurosurgical hospitals are Haaglanden Medical Centre (HMC) The Hague, Haga Teaching Hospital The Hague, Leiden University Medical Centre (LUMC) in Leiden, Medisch Spectrum Twente (MST) Enschede, Erasmus Medical Centre (EMC) Rotterdam, Isala Hospital Zwolle and University Medical Centre Groningen (UMCG). The study is open to additional participating neurosurgical centres.

### Inclusion criteria

Eligible patients must be 18 years or older and meet all of the following criteria:

1. The presence of a newly diagnosed CSDH, defined as an isodense or hypodense haematoma in the subdural space on cranial computed tomography (CT) scan. Hyperdense components may be present but must compromise less than one third of the haematoma
2. Clinical symptoms must be explained by the CSDH
3. The patient is eligible for BHC and DXM based on clinical symptoms and radiological appearance of CSDH
4. MGS grade 1–3.

The MGS is a validated grading system (score 0–4, see Table 2) for the severity of neurological symptoms and is used to classify the neurological condition for CSDH patients [17].

### Exclusion criteria

Exclusion criteria are:

1. MGS grade 0 or 4
2. An acute subdural haematoma
3. The presence of a minimal CSDH on cranial CT which is technically not drainable by BHC
4. Pregnancy
5. Cerebrospinal fluid shunt in situ (e.g. ventriculoperitoneal shunt)
6. Known hypersensitivity to DXM
7. Known ulceration in the gastro-intestinal tract
8. Poorly regulated diabetes mellitus (DM) defined as a glycosylated haemoglobin (HbA1C) value > 8% (64 mmol/mol)
9. Clinical suspicion of an acute systemic infection (fever, leucocytosis, elevated C-reactive protein)
10. History of gastro-intestinal bleeding
11. Glaucoma
12. Previous history of severe affective disorders (i.e. psychosis).

### Participant timeline

**Fig. 1:**
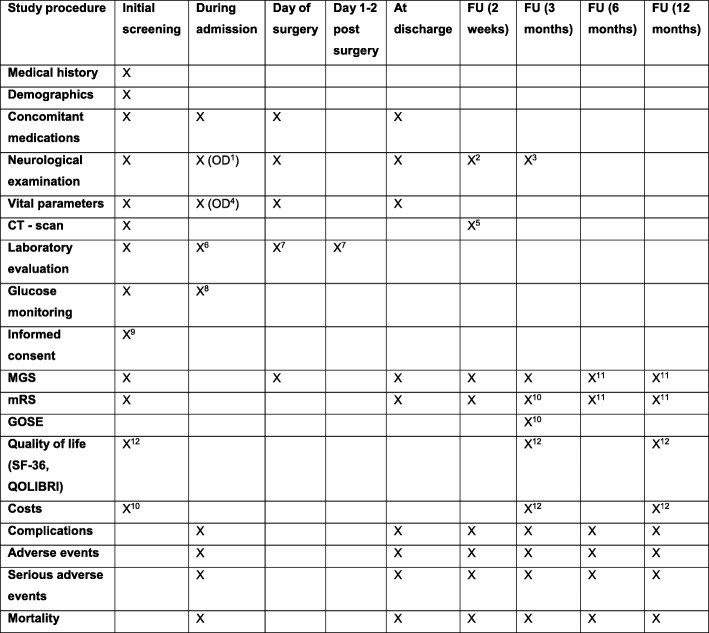
Time schedule of study procedures

**Fig. 2:**
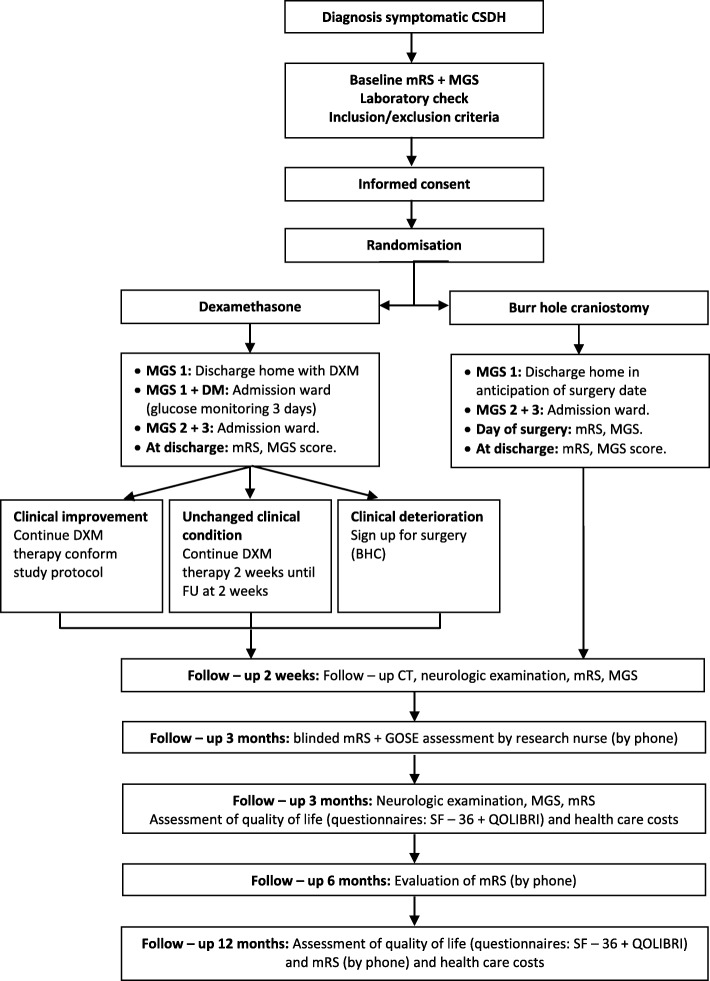
Flow diagram of main study procedures

The time schedule in Fig. 1 describes all study processes, assessments and interventions. The flow diagram (Fig. 2) displays the main study procedures, including follow-up evaluations.

In summary, study patients will be evaluated at presentation (baseline), during their hospital stay, at discharge and during the follow-up period at 2 weeks, 3 months, 6 months and 12 months.

At 2 weeks (after initiation of the study treatment) patients will be evaluated by neurological examination combined with a follow-up CT scan at the outpatient clinic or ward and at 3 months at the outpatient clinic. A mRS-certified research nurse, blinded for treatment allocation, will evaluate the primary outcome (mRS score) at 3 months by phone. At 3 and 12 months, patients will receive questionnaires on quality of life. Additionally, an evaluation of mRS score will take place by phone at 3, 6 and 12 months. Healthcare and productivity costs will be evaluated at 3 and 12 months. We expect to complete patient inclusion in 3 years. The estimated duration of the study (including follow-up) will be 4 years.

### Interventions

#### Investigational treatment

Patients in the intervention arm will receive DXM in a daily dosage of 16 mg (8 mg every 12 h) on days 1 to 4. Thereafter, DXM will be tapered by half every 3 days (see Table 4 for the DXM dosing scheme) until a dosage of 0.5 mg a day on day 19 and stopped on day 20. DXM is administered orally in tablets or intravenously when oral administration is not possible. If the patient improves on DXM therapy (defined by ≥ 1 point decrease in MGS score) during the first 2 weeks, the treatment will be continued until day 19. During DXM treatment a proton pump inhibitor (pantoprazole, 40 mg daily) is administered as prophylaxis.

**Table 4:** Dexamethasone (DXM) dosing scheme

Day	DXM dosage
1–4	8 mg every 12 h
5–7	4 mg every 12 h
8–10	2 mg every 12 h
11–13	1 mg every 12 h
14–16	0.5 mg every 12 h
17–19	0.5 mg per day
20	Stop

DXM therapy can be discontinued for the following reasons: (1) no improvement of the clinical condition, defined as an unchanged MGS score 2 weeks after initiation of DXM therapy with unchanged or increased haematoma on the follow-up CT at 2 weeks, (2) clinical deterioration, defined by as ≥ 1 point increase in MGS score, at any time after initiation of DXM treatment, (3) the occurrence of severe, relevant DXM-related side effects or complications (i.e. uncontrollable hyperglycaemia, gastro-intestinal bleeding or psychiatric symptoms), (4) pre-study complement discontinuation of DXM therapy is primarily left to the discretion of the treating physician and is recommended in case of: persistence of moderate to severe neurological symptoms (MGS grade 2–3) in combination with the presence of relevant severe, current comorbidities (i.e. an infection, metabolic deterioration) which could interfere with the expected recovery, and a surgical intervention could be beneficial and the safest option for patient recovery.

In any case of pre-study discontinuation of DXM therapy, the reason for this is documented. Whenever DXM is discontinued, a cross-over to the reference treatment (BHC) can occur depending on the remaining symptoms, which is the local standard of care in the participating hospitals.

#### Reference treatment

Patients randomised to the reference treatment arm are operated on preferably within the first 7 days, depending on anticoagulant or antithrombotic therapy use, severity of symptoms and discretion of the treating physician. Surgery will take place through BHC followed by insertion of a subdural drain for 2 days in line with the standard protocols in each participating hospital. Antibiotic prophylaxis is administered preoperatively. Either general or local anaesthesia will be applied. One or two 14-mm burr holes, depending on the surgeon’s discretion, are drilled over the maximum width of the haematoma. The dura mater is opened with a cruciate incision and coagulated with bipolar diathermy. The subdural collection is washed out with warm Ringer’s lactate saline, with or without a catheter. The subdural outer and inner membrane loculations, if present, can be disrupted when easily accessible via the burr holes. Whenever the saline has dispersed sufficiently a subdural drain is placed and the wound is closed.

Reoperation can be indicated when neurological deficits do not resolve, deteriorate or recur within the follow-up duration. Treatment options consist of redo burr-hole evacuation, if necessary, through another additional hole, percutaneous aspiration, craniotomy, or craniectomy.

#### Concomitant care

All included patients will otherwise receive routine standard of care. Patients with mild neurological deficits (MGS grade 1) on admission can be discharged home in anticipation of the planned BHC or awaiting the effect of DXM therapy. However, in MGS grade 1 patients with known diabetes mellitus (DM) with HbA1C < 64 mmol/mol randomised for DXM therapy, monitoring for blood glucose levels is necessary during the first 3 days after treatment initiation. Glucose monitoring can take place clinically during admission or if possible at the nursing home. Patients with MGS grade 2–3 (in either arm) remain in hospital until the treating physician judges the clinical situation safe for discharge.

During admission neurological investigations and vital parameters are recorded daily. Low-molecular-weight heparin (LMWH) will be applied in both patient groups as thrombosis prophylaxis if the patient is not optimally mobile. Patients will receive physiotherapy, speech therapy or rehabilitation consultation if deemed necessary.

##### Anticoagulant or antithrombotic therapy

Oral anticoagulant or antithrombotic therapy will be discontinued in both study arms from the moment of randomisation to prevent haematoma growth and to avoid interference with planned surgery. In case of vitamin K antagonist (VKA) therapy the international normalised ratio (INR) is corrected to ≤ 1.5 through the administration of vitamin K and/or prothrombin complex concentrate (PCC), as is the current practice. For patients using platelet-aggregation-inhibitor therapy, surgery is preferably planned 7 days after discontinuation of therapy, if allowed by the clinical condition. At the discretion of the surgeon, earlier intervention is allowed if deemed clinically necessary. The reason for early surgery has to be recorded in the case report form (CRF). Non-vitamin-K oral anticoagulants (NOACs) are discontinued at least 1 day prior to surgery.

Any anticoagulant or antithrombotic therapy can be resumed 2 weeks after the initiation of DXM therapy or surgery following a follow-up CT without signs of CSDH recurrence, recent-onset haematoma or unchanged mass effect with midline shift compared to the initial CT at randomisation. Partial resolution of CSDH at this stage without recent haematoma is not a contraindication for resumption. For absolute indications (e.g. mechanic cardiac valve) earlier resumption or bridging of therapy within these 14 days is allowed. Any reason for early resumption has to be recorded in the CRF. Subgroup analyses will be performed to evaluate the effect of anticoagulant therapy in both groups.

### Outcomes

#### Primary outcome measures

The primary endpoints are the functional outcome, expressed by mRS, at 3 months after start of study treatment and cost-effectiveness at 12 months.

#### Secondary outcome measures

Secondary outcomes include: functional and clinical outcome, expressed by mRS and MGS scores, respectively, at discharge, at 2 weeks, at 3, 6 and 12 months after start of study treatment. We will also determine a utility-weighted mRS (UW-mRS) at 3 months. The GOSE score will be assessed at 3 months, quality of life (expressed by SF-36 and QOLIBRI) at 3 and 12 months, cost-effectiveness at 3 and 12 months and haematoma thickness at 2 weeks. During the first 12 months, we will evaluate haematoma recurrence (defined as recurrence of symptoms and neurological signs after initial improvement with persistence, recurrence or increase of CSDH on follow-up CT), failure of therapy after randomisation and requiring intervention, complications and drug-related adverse events, duration of hospital stay and healthcare and productivity costs in both patient groups. Finally, we will evaluate mortality during the first 3 and 12 months.

### Randomisation

Patients are randomised in a 1:1 allocation ratio stratified for study site by their treating physician. Stratified block-randomisation is done by using a computer randomisation algorithm to generate balanced random samples (Castor EDC, Ciwit B.V., Amsterdam, The Netherlands).

### Sample size

This RCT is designed as a non-inferiority study. The sample size for showing non-inferiority is calculated based on a simulation programme in R statistical software for power for ordinal regression. We aim to include 420 patients. This sample size yields a power of 90%, assuming that the true effect of DXM is an odds ratio 1.15 for a better functional outcome on the mRS, and the limit for inferiority is an odds ratio < 0.9.

### Data collection

All patient data is collected in the electronic data capture software Castor EDC (Ciwit B.V., Amsterdam, The Netherlands). This software allows built-in logical checks and validations to promote data quality. Data entry is performed locally by trained research nurses and physicians. No patient-identifying information is collected.

### Data analysis

The primary effect parameter (and all other comparisons of the treatment arms) will be performed on all randomised subjects according to the intention-to-treat (ITT) principle. A sensitivity analysis is performed for the primary outcome measure in a per-protocol fashion, defined as patients in the ITT population receiving treatment as randomised without protocol violation.

The primary effect parameter will be the adjusted common odds ratio (acOR) for a shift in the direction of a better outcome on the mRS at 3 months with 95% confidence interval, estimated with multivariable ordinal logistic regression with adjustment for important prognostic baseline variables. This analysis is becoming the standard for ordinal functional outcomes in neurology and neurosurgery, supported by evidence for its maximisation of statistical power while maintaining interpretability. Missing data in baseline characteristics will be imputed using multiple imputation (*n* = 10) based on the outcome and relevant baseline covariates using the ‘Multivariate Imputation by Chained Equations’ (MICE) algorithm. Patients with missing primary outcome will be excluded but every effort will be made to obtain follow-up. To accept the null hypothesis (H0) of non-inferiority the lower 95% confidence limit of the odds ratio for a better functional outcome on the mRS of DXM versus surgery should be equal to or above 0.9.

Furthermore, we will perform an extensive economic evaluation of DXM versus surgery for patients with a CSDH. The economic evaluation will be performed according to the Dutch guidelines, using a societal perspective. The timeframe will be 12 months to take all relevant costs and effects into account. The primary effect measure for the economic evaluation will be functional status (mRS). Secondary outcome measures for the cost-effectiveness analyses (CEA) will be mortality and quality-adjusted life year (QALY), based on the 12-month SF-36 and QOLIBRI summary scores. The cost-effectiveness will be assessed by calculating the incremental cost-effectiveness ratio (ICER), defined as the difference in costs, divided by the average change in effectiveness of DXM versus surgery in CSDH patients. The cost-effectiveness analysis will use the mRS as effect measure and the cost-utility analysis will use the QALY as effect measure.

Uncertainty around this ratio will be presented using confidence eclipses on the cost-effectiveness plane and acceptability curves. We will perform a sensitivity analysis to assess the robustness of the results to changes in costs and effectiveness parameters. Due to the short time horizon, no discounting for costs and effects will be used (see Additional file 2 for statistical analysis plan).

For secondary endpoint parameters, Kaplan-Meier and Cox regression analysis will be used for mortality comparisons between the treatment arms, binary logistic regression for complications and failure of therapy, and a linear regression to evaluate quality of life. A *p* value of less than 0.05 will be used to indicate statistical significance. For all analyses, R statistical software will be used.

### Study monitoring

#### Data monitoring

The coordinating investigator will visit study centres every 3 months to discuss any issues and check on conduct of the study. Prior to recruitment, the field team (physicians) will receive information and instructions on the objectives of the study, methods and processes of the study. CRF data will be monitored by an independent external expert at regular intervals throughout the study to verify adherence to the protocol and data completeness, consistency and accuracy.

#### Data Safety Monitoring Board (DSMB)

In order to increase the safety of the intervention the trial will be monitored by an independent Data Safety Monitoring Board (DSMB). The DSMB will work in accordance with a dedicated charter and will follow processes recommended by the DAMOCLES Statement. The DSMB will be chaired by a neurosurgeon, and include a neurologist and an independent methodologist/statistician. The DSMB will meet at least annually or after inclusion of the next 150 patients (whichever comes first). With respect to study safety and efficacy, interim analyses of major endpoints (including serious adverse events believed to be due to treatment) are performed after 150 and 300 patients have completed their follow-up evaluation. In addition, the DSMB will review the study logistics/trial conduct in terms of: assessment of compliance with the study protocol (including adherence to inclusion and exclusion criteria) and monitor data quality (completeness), time to start of the procedure (DXM/surgery), cross-overs, occurrence and listing/registration of (serious) adverse events, by centre and by treatment arm.

#### Adverse events (AEs) and serious adverse events (SAEs)

Adverse events are defined as any undesirable event occurring to a patient during the study, whether or not considered related to DXM therapy or surgery. All adverse events reported spontaneously by the patient or observed by the investigator or staff will be recorded. A SAE is any untoward medical occurrence or effect that results in death; is life-threatening (at the time of the event); requires hospitalisation or prolongation of existing inpatients’ hospitalisation; results in persistent or significant disability or incapacity or any other important medical event that did not result in any of the outcomes listed above due to medical or surgical intervention, but could have been based upon appropriate judgement by the investigator. SAEs are reported by the investigators in participating centres to the coordinating investigator. SAEs will be reported through the web portal *ToetsingOnline* to the accredited Medical Ethics Committee that approved the protocol.

#### Interim analysis

Interim analyses of major endpoints (including serious adverse events believed to be due to treatment) are performed after 150 and 300 patients have completed their follow-up evaluation.

### Dissemination of results

Trial results will be published in an international journal, communicated to neurological and neurosurgical associations and presented at (inter)national congresses.

## Discussion

General guidelines defining the preferred treatment for CSDH are lacking, but worldwide, surgery is the current standard practice. Various hospitals, however, apply DXM as an alternative treatment modality or as an adjunctive therapy prior to surgery. To date, no head-to-head trial comparing the two modalities in a well-defined cohort of patients has been performed to our knowledge. The competing benefit of either treatment is, therefore, not clear.

CSDH development occurs likely due to (mild) traumatic brain injury causing a tear in the dural border cell layer which leads to extravasation of cerebrospinal fluid and blood in the subdural space. At a point neurological deficits arise because of a mass effect due to liquefaction and progressive enlargement of an initially small haematoma. The rationale behind corticosteroid therapy is based on results of previous experimental work that postulates an inflammatory response to be responsible for the haematoma enlargement [13, 14, 18–23]. Accumulated blood in the subdural space, in particular erythrocyte breakdown products, incites an inflammatory reaction that results in the deposition of fibrin and formation of subdural neo-membranes with in-growth of neo-capillaries. These neo-membranes are vulnerable structures with high vascularisation of the outer layer and are prone to rupture and bleed. Furthermore, it is also believed that the outer layer of the neo-membrane contains a high content of plasminogen and plasminogen activator, which cause an enzymatic fibrinolysis and liquefaction of the initial blood clot in the inner haematoma. This situation finally results in frequent effusions of plasma or rebleeding from the neo-membranes into the subdural collection. Hence, a cascade of inflammation, impaired coagulation, angiogenesis and fibrinolysis plays an important role in the formation of CSDH.

Despite this dynamic hypothesis regarding the pathophysiology of CSDH, high-quality data supporting the use of DXM therapy as alternative treatment to surgery is scarce. Previous studies have shown favourable results of DXM as adjunctive to surgery in reducing mortality [24] and reoperation rate [15, 25, 26]. In current literature only four (non-randomised) studies evaluated the effect of corticosteroids in CSDH management as monotherapy compared to corticosteroids as an adjunctive to surgery or surgery alone [15]. In each study a different primary outcome measure was applied, of which only two used a validated outcome scale to assess functional outcome.

Of these two studies, the first study had a prospective design and evaluated 112 patients in four patient groups: DXM monotherapy, DXM in combination with surgery by BHC without additional drainage, surgery only and observation only [27]. This study revealed a favourable outcome, defined by a Glasgow Outcome Scale (GOS) score of 4–5 at 6 months, in 88% after DXM monotherapy. The reported success rate (GOS 4–5) for DXM therapy adjunctive to surgery was 91%, compared to 77% after surgery alone and 50% after observation only. The second study described a retrospective evaluation in 122 patients in slightly different patient groups: initial DXM therapy, surgery alone by twist-drill mini-craniostomy, surgery alone by craniotomy and observation only [28]. A favourable outcome was expressed by a Markwalder Grading Scale (MGS) score of 0–2 at discharge and was achieved in 73% after DXM monotherapy. In 25% of patients receiving initial DXM therapy, monotherapy failed and additional surgery was required in this group. In the primary surgical groups the reported success rates (MGS score 0–2) were 93 and 75% after twist-drill mini-craniostomy and craniotomy, respectively, and for the observation only group 100%.

In contrast, extensive research has been performed regarding the several operative techniques. Different surgical techniques can be applied, such as craniotomy, BHC or twist-drill craniostomy, with or without placement of a subdural drain. To date, no class I evidence exists to compare the various methods of haematoma evacuation. A recent large systematic review evaluated all 24 available RCTs regarding surgical treatment of CSDH. The only significant finding was a reduction in haematoma recurrence after postoperative subdural drainage based on eight RCTs [9]. In addition, one of the largest RCTs, performed in 215 symptomatic CSDH patients, showed that subdural drainage compared to no drainage not only lowered recurrence rate, but also reduced mortality and improved functional outcome at 6 months [10].

Overall, surgical techniques have been thoroughly demonstrated as effective therapy in the current literature for CSDH patients. DXM is showing promising results as an alternative treatment, but confirmation of these results is essential by means of RCTs.

### Trial status

This trial started on 1 September, 2016. The first patient was included in Medical Centre Haaglanden (HMC) The Hague and subsequently enrolment was started in Haga Teaching Hospital The Hague and Leiden University Medical Centre (LUMC) Leiden. The trial will start on 1 August 2018 at Erasmus Medical Centre (EMC) and Medisch Spectrum Twente (MST) and on 1 September 2018 at Isala Hospital Zwolle and Groningen University Medical Centre (UMCG). The study is open to additional participating neurosurgical centres.

## Additional files

Additional file 1: Standard Protocol Items: Recommendations for Interventional Trials (SPIRIT) Checklist. (PDF 181 kb)

Additional file 2: Statistical analysis plan. (PDF 253 kb)

## Abbreviations

BHC: Burr-hole craniostomy
CEA: Cost-effectiveness analyses
CSDH: Chronic subdural haematoma
CT: Computed tomography
DM: Diabetes mellitus
DSMB: Data Safety Monitoring Board
DXM: Dexamethasone
EMC: Erasmus University Medical Centre
GOS: Glasgow Outcome Scale
GOSE: Glasgow Outcome Scale-Extended
HMC: Haaglanden Medical Centre
INR: International normalised ratio
LUMC: Leiden University Medical Centre
METC: Medical Ethics Committee
MGS: Markwalder Grading Scale
MRS: Modified Rankin Scale
MST: Medisch Spectrum Twente
NOAC: New oral anticoagulant
OD: Once a day
PCC: Prothrombin complex concentrate
QOLIBRI: Quality of Life in Brain Injury
RCT: Randomised controlled trial
SF-36: Short Form – 36 Health Survey
UMCG: University Medical Centre Groningen
VKA: Vitamin K antagonist
ZonMw: Netherlands Organisation for Health Research and Development
