# Supplementary material for: Dexamethasone therapy versus surgery for chronic subdural haematoma (DECSA trial): study protocol for a randomised controlled trial
Source: Trials. 2018 Oct 20;19:575. doi: 10.1186/s13063-018-2945-4 (PMC6196013; doi:10.1186/s13063-018-2945-4)
Supplement: Supplementary file 2 — Statistical analysis plan. (PDF 253 kb) [file 13063_2018_2945_MOESM2_ESM.pdf]

STATISTICAL ANALYSIS PLAN (SAP)

Dexamethasone Therapy versus Surgery for Chronic Subdural Haematoma, a clinical randomised controlled trial (DECSA – trial)

Statistical Analysis Plan (SAP)

Version: 1.0

Authors:

- Ishita P. Miah MD, resident Neurology, Haaglanden Medical Centre (HMC) The Hague, The Netherlands.
- Hester F. Lingsma, epidemiologist department of Public Health, Erasmus MC University Medical Center, Rotterdam, The Netherlands

Date: 01-07-2018

1. INTRODUCTION

The purpose of the Dexamethasone Therapy versus Surgery for Chronic Subdural Haematoma Chronic subdural haematoma (CSDH) – trial (DECSA) is to evaluate non-inferiority of primary dexamethasone (DXM) therapy compared to primary burr-hole craniostomy (BHC) on functional outcome and to compare the cost-effectiveness of these two treatments in patients with symptomatic CSDH.

Here we will summarize the planned statistical analysis of the data. We will describe how missing data will be handled and how analyses of the primary and secondary outcome measures will be performed.

2. DATA SOURCE

The DECSA trial is multicentre clinical trial with randomized treatment allocation with open label treatment and blinded endpoint evaluation. Patients aged 18 years or older with a symptomatic CSDH with a Markwalder Grading Score [1] 1-3, fulfilling the remaining inclusion and exclusion criteria as described below in section 4, are eligible patients for the study. Patients are randomised to one of the two treatment arms: DXM therapy (the intervention arm) or BHC (control arm).

Patients will be recruited for the study from the emergency department, neurological or neurosurgical outpatient clinic or ward or through referral from general hospitals of the seven participating Dutch neurosurgical hospitals. The seven participating neurosurgical hospitals are Haaglanden Medical Centre (HMC) The Hague, Haga Teaching Hospital The Hague, Leiden University Medical Centre (LUMC) in Leiden, Medisch Spectrum Twente (MST) Enschede, Erasmus Medical Centre (EMC) Rotterdam, Isala Hospital Zwolle and University Medical Centre Groningen (UMCG). The study is open to additional participating neurosurgical centres.

3. ANALYSIS OBJECTIVES

The primary objective is to evaluate the non-inferiority of primary DXM therapy versus primary BHC on functional outcome as expressed by the modified Rankin Scale (mRS) score [2] (table 1) at three months and to compare the two treatments in terms of cost-effectiveness, expressed by costs per quality adjusted life year measured with the Short Form – 36 Health Survey (SF–36) [4] and Quality of Life after Brain Injury Overall Scale (QOLIBRI) [5] at twelve months, in patients with symptomatic CSDH.

Table 1. Modified Rankin Scale (mRS)

Score	Functional status
0	No symptoms.
1	No significant disability. Able to carry out all usual activities, despite some symptoms.
2	Slight disability. Able to look after own affairs without assistance, but unable to carry out all previous activities.
3	Moderate disability. Requires some help, but able to walk unassisted.
4	Moderately severe disability. Unable to attend to own bodily needs without assistance, and unable to walk unassisted.
5	Severe disability. Requires constant nursing care and attention, bedridden, incontinent.
6	Dead.

4. ANALYSIS SETS/ POPULATIONS/SUBGROUPS

Analysis will be performed in patients participating the study and fulfilling the following inclusion and exclusion criteria:

Inclusion criteria

Eligible patients must be 18 years or older and meet all of the following criteria:

1) presence of a newly diagnosed CSDH
2) clinical symptoms must be explained by the CSDH
3) patient is eligible for BHC and DXM based on clinical symptoms and radiologic appearance of CSDH
4) Markwalder Grading Scale (MGS) score 1 – 3
5) Written informed consent.

The MGS is a validated grading system (grade 0-4, see table 2) for severity of neurological symptoms and is used to classify the neurological condition for CSDH patients.

Exclusion criteria

1) MGS 0 or MGS 4
2) an acute subdural haematoma
3) presence of a minimal CSDH on cranial CT which is technically not drainable by BHC
4) pregnancy
5) cerebrospinal fluid shunt in situ (e.g. ventriculoperitoneal shunt)
6) known hypersensitivity to DXM
7) known ulceration in the gastro-intestinal tract
8) poorly regulated diabetes mellitus (DM) defined as a HbA1C value >8% (64 mmol/mol)
9) clinical suspicion of an acute systemic infection (fever, leucocytosis, elevated C-reactive protein)
10) history of gastro-intestinal bleeding,
11) glaucoma
12) previous history of severe affective disorders (i.e. psychosis).

5. ENDPOINTS

Primary endpoints:

- mRS at three months.
- Cost – effectiveness at twelve months.

Secondary endpoints:

- Functional and clinical outcome, expressed by mRS and MGS score respectively at discharge, at two weeks, three, six and twelve months and the Glasgow Outcome Scale – Extended (GOSE) score [3] (table 3) at three months.
- Haematoma thickness at two weeks on follow–up CT
- Mortality during the first three and twelve months.
- Haematoma recurrence during the first twelve months.
- Complications and drug related adverse events during the first twelve months.

- Failure of therapy after randomisation and requiring intervention within the first twelve months.
- Duration of hospital stay during the first twelve months.

Table 2. Markwalder Grading Scale

Score	Clinical status
0	Patient neurological normal.
1	Patient alert and oriented; mild symptoms such as headache; absent of mild neurological deficit such as reflex asymmetry.
2	Patient drowsy (defined as Glasgow Coma Scale, GCS, score: 13-14) or disoriented with variable neurological deficit, such as hemiparesis.
3	Patient stuporous (defined as GCS 9 – 12) but responding appropriately to noxious stimuli; severe focal signs such as hemiplegia.
4	Patient comatose (GCS 8 or lower) with absent motor responses to painful stimuli; decerebrate or decorticate posturing.

Table 3. Glasgow Outcome Scale – Extended

Score	Category	Score	Category
**1**	Death	**5**	Moderate disability, lower
**2**	Vegetative state	**6**	Moderate disability, upper
**3**	Severe disability, lower	**7**	Good recovery, lower
**4**	Severe disability, upper	**8**	Good recovery, upper

6. HANDLING OF MISSING VALUES AND OTHER DATA CONVENTIONS

Missing data in baseline characteristics will be imputed using multiple imputation (n=10) based on the outcome and relevant baseline covariates using the ‘Multivariate Imputation by Chained Equations’ (MICE) algorithm. Patients with

missing primary outcome will be excluded but every effort will be made to obtain follow-up.

7. STATISTICAL METHODOLOGY

7.1 STATISTICAL PROCEDURES

The primary analysis (and all other comparisons of the treatment arms) will be performed on all randomized subjects according to the Intention-To-Treat (ITT) principle.

The primary effect parameter will be the adjusted common odds ratio (acOR) for a shift in the direction of a better outcome on the mRS at 3 months with 95% confidence interval, estimated with multivariable ordinal logistic regression with adjustment for important prognostic baseline variables as age, sex, MGS, mRS and GOSE score at baseline, duration between start of symptoms and initiation of treatment, cardiovascular risk factors (diabetes mellitus, atrial fibrillation, hypertension, stroke, myocardial infarction, venous thrombosis) and coagulopathy. To accept the null hypothesis (H0) of non-inferiority the lower 95% confidence limit of the odds ratio for a better functional outcome on the mRS of DXM versus surgery should be equal to or above 0.9.

Furthermore, we will perform an extensive economic evaluation of DXM versus surgery for patients with a CSDH. The economic evaluation will be performed according to the Dutch guidelines, using a societal perspective. The timeframe will be 12 months to take all relevant costs and effects into account. The primary effect measure for the economic evaluation will be functional status (mRS). Secondary outcome measures for the CEA will be mortality and QALY, based on the 12-month SF-36 and QOLIBRI summary score. The cost-effectiveness will be assessed by calculating the incremental cost-effectiveness ratio (ICER), defined as the difference in costs, divided by the average change in effectiveness of DXM versus surgery in CSDH patients. The cost-effectiveness analysis will use the mRS as effect measure and the cost-utility analysis will use QALY as effect measure.

Uncertainty around this ratio will be presented using confidence eclipses on the cost-effectiveness plane and acceptability curves. We will perform a sensitivity analysis to assess the robustness of the results to changes in costs and effectiveness parameters. Due to the short time horizon, no discounting for costs and effects will be used.

For secondary endpoint parameters, Kaplan-Meier and Cox regression analysis will be used for mortality comparisons between the treatment arms, binary logistic regression for complications and failure of therapy, and a linear regression to evaluate Quality of Life. A p-value of less than 0.05 will be used to indicate statistical significance. For all analyses, R statistical software will be used.

7.2 MEASURES TO ADJUST FOR MULTIPLICITY, CONFOUNDERS, HETEROGENEITY, ETC.

Not applicable as this is an RCT. Covariate adjustment will be performed to increase statistical power as described above.

8. SENSITIVITY ANALYSES

A sensitivity analysis is performed for the primary-outcome measure in a per-protocol fashion, defined as patients in the intention-to-treat population receiving treatment as randomised without protocol violation.

12. REFERENCES

1. Markwalder T, Steinsiepe KF, Rohner M, Reichenbach W, Markwalder H. The course of chronic subdural haematomas after burr-hole craniostomy and closed-system drainage. J Neurosurg 1981;55:390-396.
2. Banks JL, Marotta CA. Outcomes validity and reliability of the modified rankin scale: implications for stroke clinical trials. A literature review and synthesis. Stroke 2007;38:1091-1096.
3. Weir J, Steyerberg EW, Butcher I, Lu J, Lingsma HF, McHugh GS et al. Does the extended Glasgow outcome scale add value to the conventional Glasgow outcome scale. J Neurotrauma 2012;29:53-58.
4. Ware JE, Sherbourne CD. The MOS 36-item short-form health survey (SF-36) I. Conceptual framework and item selection. Med Care 1992;30:473-483.
5. Von Steinbuchel N, Wilson L, Gibbons H, Hawthorne G, Schmidt S, Bullinger M et al. Quality of life after brain injury (QOLIBRI): scale validity and correlates of quality of life. J Neurotrauma 2010;27:1157-1165.

13. APPENDICES

Not applicable.
